# Case report: High-grade hidradenocarcinoma of the chest wall with insights from ^18^F-FDG PET/CT imaging and a review of the literature

**DOI:** 10.3389/fonc.2024.1493232

**Published:** 2024-12-16

**Authors:** Wenxin Li, Xianwen Hu, Na Tan, Pan Wang

**Affiliations:** ^1^ Department of Nuclear Medicine, Affiliated Hospital of Zunyi Medical University, Zunyi, China; ^2^ Department of Pathology, Affiliated Hospital of Zunyi Medical University, Zunyi, China

**Keywords:** hidradenocarcinoma, chest, high-grade, pathology, PET/CT

## Abstract

Hidradenocarcinoma (HAC) is a rare neoplasm that typically occurs in the head and neck region but seldom affects the chest wall. Histopathology and immunohistochemistry remain essential for diagnosing HAC, although their clinical utility in determining metastasis can be limited. Given the pathological rarity and histopathological heterogeneity of HAC, we report a case demonstrating the utility of positron emission tomography/computed tomography (PET/CT) combined with immunohistochemical examination for the accurate diagnosis and staging of HAC. An 84-year-old woman presented to our hospital with a right chest wall and axillary mass. A pathological examination was performed, which revealed a malignancy of epithelial origin. The immunohistochemical examination confirmed a high-grade hidradenocarcinoma. Subsequently, PET/CT examination showed significant hypermetabolism in the right chest wall and its ipsilateral axillary and subclavian lymph nodes. Combined with pathological findings, these results confirmed metastatic hidradenocarcinoma, leading to a TNM classification of T2N3M (stage IV). A literature review revealed that HAC rarely occurs in the chest wall but tends to metastasize. However, the prognosis is favorable, especially with early diagnosis and surgical intervention. ^18^F-FDG PET/CT examination is a valuable staging tool in the comprehensive assessment of systemic tumor metastasis. Combining PET/CT with pathological examination enhances diagnostic and staging accuracy, enabling timely treatment and improving outcomes.

## 1 Introduction

Primary malignant hidradenoma or hidradenocarcinoma (HAC) is a rare form of cancerous adnexal neoplasm originating from the eccrine sweat glands. It accounts for only 0.001% of all tumors and 6% of all malignant eccrine tumors, with an incidence of only 0.05% in the United States (1, 2). HAC typically affects individuals aged ≥50 years, although it can occur at any age (3). It is most commonly found in the head and neck region, with only a small proportion occurring in the chest wall (4). Compared with its benign counterpart, HAC is highly susceptible to metastasis, invasion, and recurrence (5). Owing to its rarity, studies related to this tumor are limited, and most of them are presented as isolated small case series, making its characterization and management challenging and at times contradictory. For example, a previous study suggested an extremely poor prognosis for HAC; however, Teng et al. concluded that the prognosis of HAC is generally favorable based on the results of a statistical analysis of 289 patients with HAC from the Surveillance, Epidemiology, and End Results database. The mean cancer-specific survival (CSS) and overall survival (OS) of HAC were 165.9 months and 164 months, respectively. Meanwhile, the 10-year CSS and OS rates were 90.5% and 60.2%, respectively (6). The lack of consensus highlights the need for more detailed HAC case reports. As a supplementary characterization method to regular histopathology and immunohistochemistry (6), fluorine-18-labeled deoxyglucose positron emission tomography/computed tomography (^18^F-FDG PET/CT) examination plays an important role in the staging and evaluation of a variety of solid tumors. In contrast, its application in the staging of HAC has been rarely demonstrated to date. Herein, we present the case of a patient with right chest wall HAC who underwent PET/CT to enhance our understanding of this rare tumor, alongside a brief review of the relevant literature.

## 2 Case description

An 84-year-old woman presented to our hospital 6 months prior to the study with a mass on her right chest wall and axilla. The chest wall mass was initially identified 2 years ago, which gradually enlarged over time without causing pain. However, 8 months ago, the mass further increased in size, and a painful axillary mass subsequently developed. The patient had a documented history of hypertension for >2 years, which was effectively managed with oral antihypertensive medications. No other significant medical or family history of malignancy was reported.

A month ago, an ultrasound examination performed at another hospital showed a well-defined subcutaneous mass on the right chest wall, measuring approximately 2.9 × 2.7 cm. The mass exhibited mixed echogenicity and blood flow signals (no clear image available) and was initially considered benign. Subsequently, an excisional biopsy at our hospital revealed a solitary chest wall mass measuring 4.5 × 3.5 × 3 cm and multiple axillary masses ranging from 2 to 7 cm.

**Figure 1:**
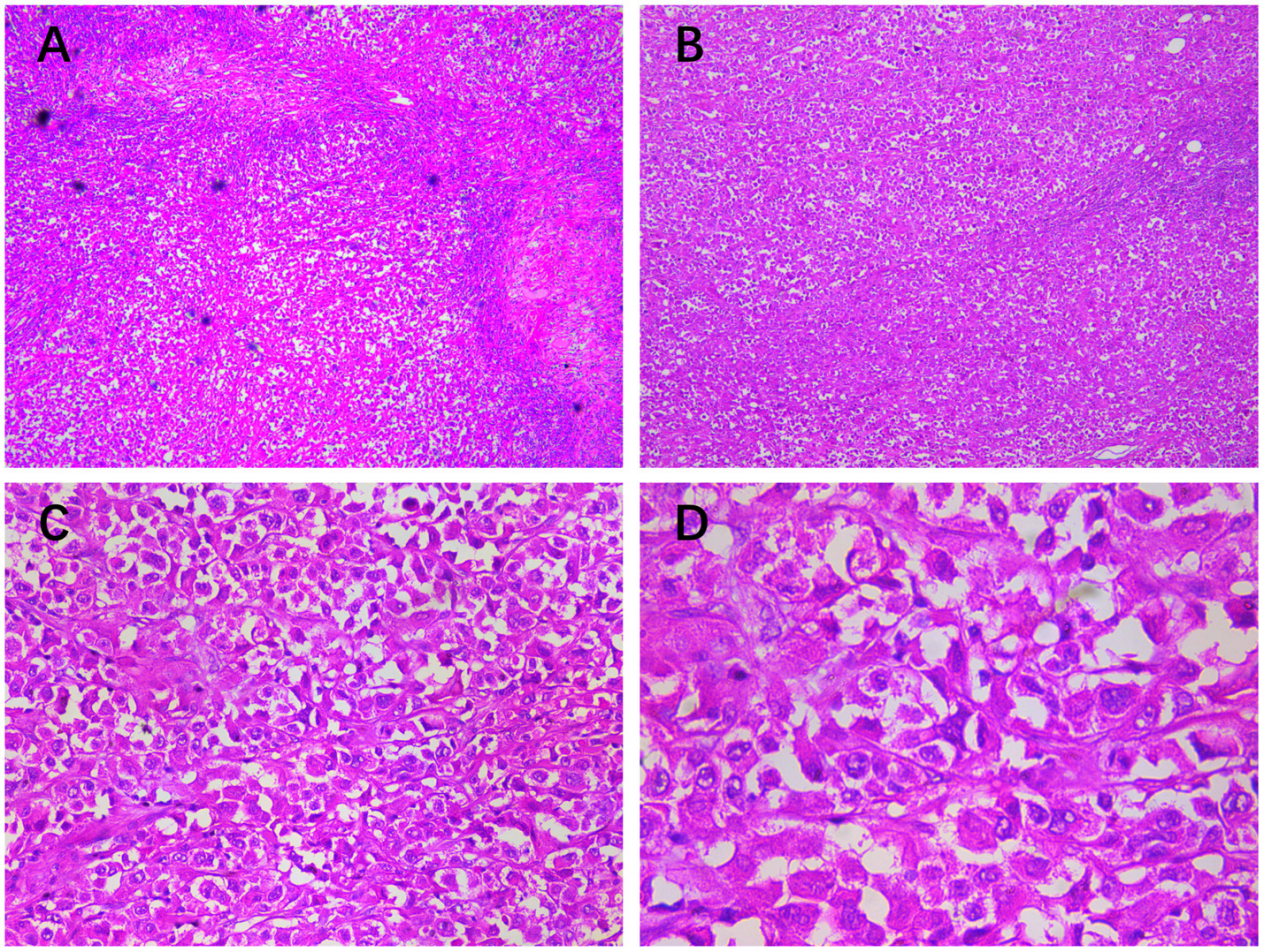
Histopathological findings (HE staining of the chest wall mass): (**A**, ×10) a large number of atypical cells were found under the epidermis in diffuse or small nests, showing infiltrative growth, fibrous septum, and (**B**, ×50) local necrosis. (**C**, ×200) The tumor cells were composed of clear cells, squamous cells, eosinophilic cells, mucinous cells, eosinophilic polygonal cells, and transitional cells in different proportions, but mainly clear cells or squamous cells with poorly differentiated atypia. (**D**, ×400) The tumor nuclei were large and hyperchromatic; some cells had visible nucleoli, increased mitotic activity, and abundant, partially eosinophilic cytoplasm; and metastatic tumor deposits in axillary lymph nodes had histological features similar to the malignant part of the primary lesion.

Histopathological examination confirmed that the mass was malignant, which was characterized by atypical cells arranged in diffuse or small nests with infiltrative growth, fibrous septa, and local necrosis. The tumor cells were composed of clear, squamous, eosinophilic, mucinous, eosinophilic polygonal, and transitional cells in varying proportions, with clear or squamous cells exhibiting poorly differentiated atypia. The tumor nuclei appeared large and hyperchromatic, with some cells showing visible nucleoli, increased mitotic activity, and abundant, partially eosinophilic cytoplasm. Metastatic tumor deposits in the axillary lymph nodes exhibited histological features consistent with the malignant component of the primary lesion (**Figure 1**).

**Figure 2:**
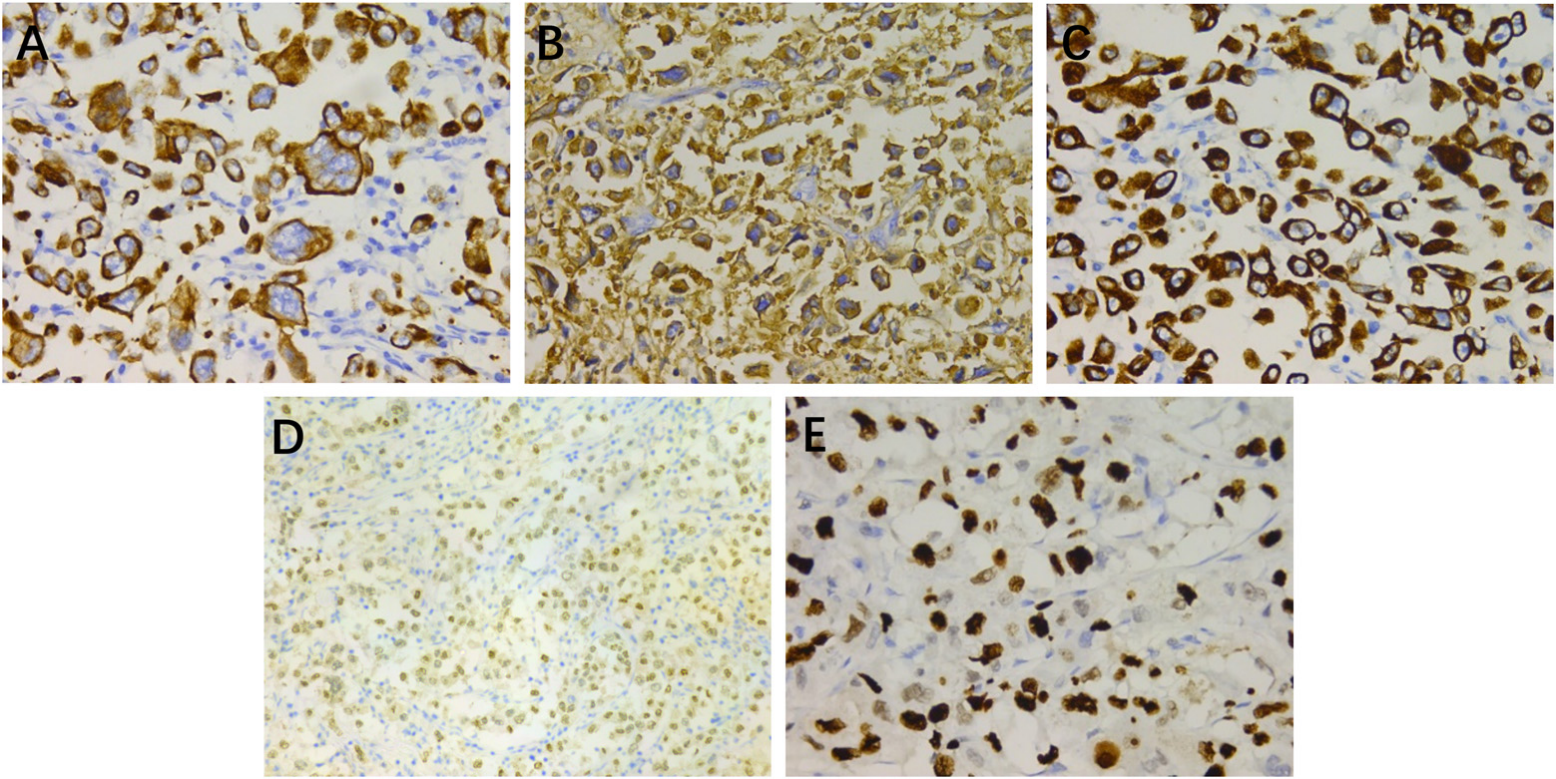
Immunohistochemical staining: **(A)** cytokeratin (×400), **(B)** epithelial membrane antigen (×400), **(C)** cytokeratin 7 (×400), **(D)** androgen receptor (×200), and **(E)** Ki-67 (×400).

Immunohistochemical examination (**Figure 2**) revealed positive staining for cytokeratin (CK), epithelial membrane antigen (EMA), cytokeratin 7 (CK7), androgen receptor (AR), with weak positivity for endothelial transcription factor 3 (GATA3) and gross cystic disease fluid protein 15 (GCDFP-15). The Ki-67 index was 80%. Staining for vimentin, CD117, cytokeratin 5/6 (CK5/6), tumor protein p63 (p63), carcinoembryonic antigen (CEA), and S100 yielded a negative result. These findings confirmed the diagnosis of high-grade HAC.

**Figure 3:**
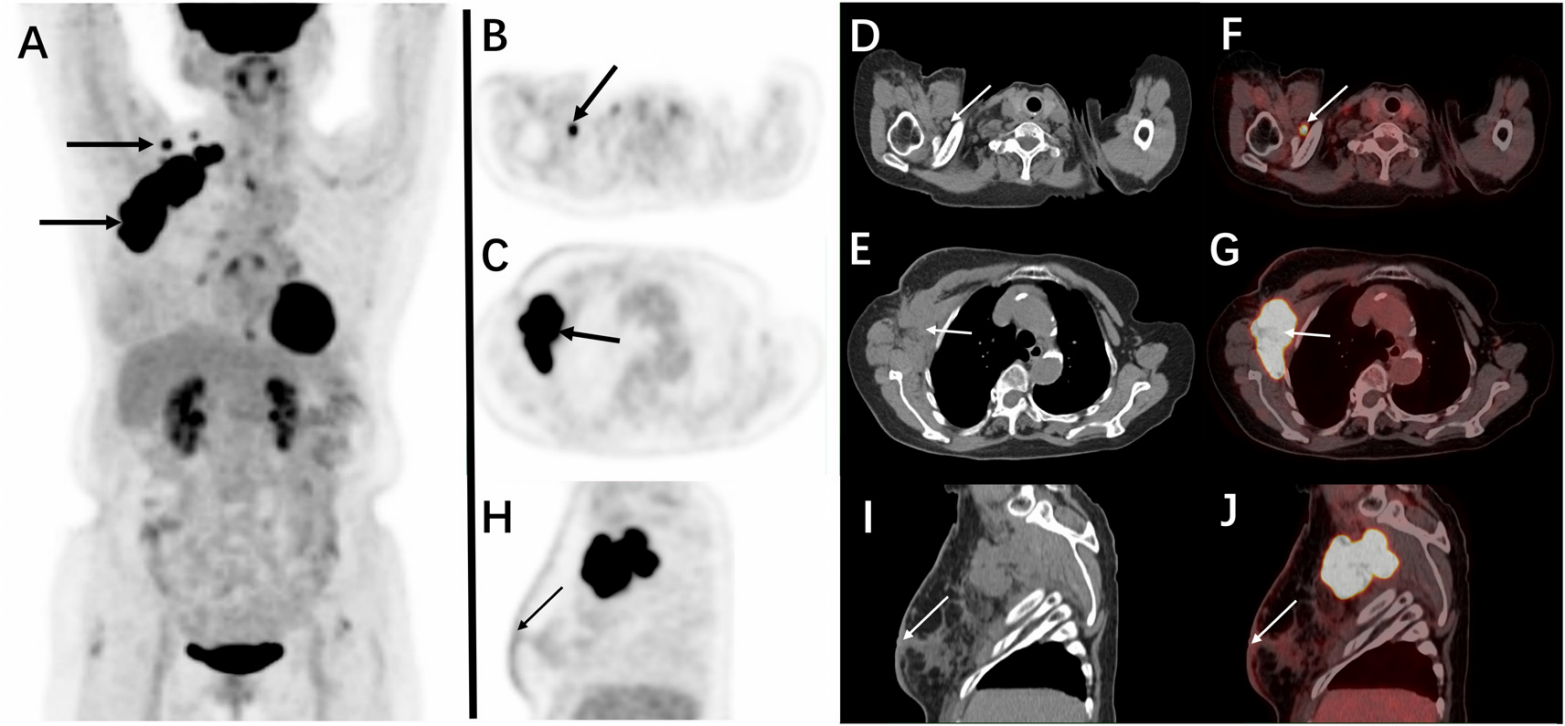
**(A)** A three-dimensional maximum intensity projection reconstruction of the PET images is seen and shows abnormal FDG uptake in the primary malignancy in the right chest wall; the SUVmax was 23.8. **(B)** Axial PET images showing the subclavian lymph nodes showed emission concentration; **(C)** radioactivity was concentrated in the chest wall and axillary lymph nodes; and the tumor developed metastasis. **(D, E)** The corresponding lesions on non-contrast CT. **(F, G)** The fused PET/CT images. **(H)** Sagittal PET images: increased size of the right breast, skin thickening, dense glands, and slightly increased metabolism; the SUVmax was 2.3. **(I)** The corresponding lesions on non-contrast CT. **(J)** The fused PET/CT images.

Subsequently, ^18^F-FDG PET/CT (**Figure 3**) revealed significant hypermetabolism in the right chest wall and ipsilateral axillary and subclavian lymph nodes. The largest section measured 5.8 × 4.8 cm with an unclear boundary. The surrounding fat space appeared fuzzy, and multiple flocculent areas of increased density were observed. The maximum standardized uptake value (SUVmax) was 23.8. The fused PET/CT image also showed an increase in right breast size, skin thickening, dense gland tissue, and slightly elevated metabolic activity, suggestive of secondary changes, although the possibility of tumor involvement could not be excluded. Based on these findings, the diagnosis was confirmed as HAC metastasis from the right chest wall to the axillary and subclavian lymph nodes (T2N3M0, stage IV).

Although clinicians proposed various treatment options, the patient declined further intervention. Over a follow-up period of 6 months, she only received morphine for pain management. During this period, her condition deteriorated, with new masses appearing in her right arm and worsening pain, suggesting further metastasis. Despite this progression, the patient refused additional treatment.

## 3 Literature review

To the best of our knowledge, no comprehensive literature review has been conducted on HAC initially discovered in the chest wall. Due to the complexities in the classification and nomenclature of skin tumors, especially those related to rare diseases, the majority of existing literature mainly consists of case series. This may lead to some synonymy when reviewing the literature, which should be noted (7). According to the National Institutes of Health Genetic and Rare Diseases Information Center, HAC is currently recognized by several synonymous terms, including “clear cell eccrine carcinoma,” “malignant acrospiroma,” “malignant clear cell acrospiroma,” “malignant nodular/clear cell hidradenoma,” and “primary mucoepidermoid cutaneous carcinoma.” Using “hidradenocarcinoma” and “chest wall” as keywords, with their respective synonyms connected by “OR,” 29 relevant articles were retrieved from PubMed and 52 from Embase. Of these articles, 11 duplicates, 6 without available full text, 26 with incomplete information, and 29 unrelated to HAC of the chest wall were excluded. Hence, only nine articles were included in the final analysis (**Table 1**) (3, 8–15).

**Table 1:** Clinical and immunohistochemical features of the cases of chest wall hidradenocarcinoma from the literature review.

Case, no.	Author, publication year, journal/book	Gender/age	Disease location	Symptom	Follow-up [months]	Metastasis	DG	TNM	Stage	AE	IHC															
											CK	CK-HMW	CK5	CK7	EMA	CEA	Ki-67	S100	AR	PR	ER	EGFR	HER-2/neu	p63	p53	PAS
1	Zhang J, 2021, Front Oncol	53/M	The inner side of the left nipple	(−)	Alive [4]	(−)	L	T2N0M0	II	HFUS, CDFI, CEUS	–	–	–	(+)	(+)	(+)	5%–30%	(±)	–	–	–	–	–	(−)	–	–
2	See SC, 2021, Pediatr Dev Pathol	20/F	Chest wall	–	–	–	L	T1N?M?	–	–	–	–	–	–	–	–	20%–25%	–	–	–	–	–	–	–	–	–
3	Brasileiro A, 2014, Indian J Dermatol Venereol Leprol	49/M	Right parasternal area	(−)	–	(−)	–	T2N0M0	II	–	(+)	–	–	–	(−)	(−)	–	–	–	–	–	–	–	–	–	–
4	Mitamura Y, 2014, Dermatol Surg	46/F	Left chest	(−)	Alive [5]	(+)	–	T2N1/2M1	IV	–	(+)	–	–	(+)	(+)	(+)	(+)	–	–	–	–	–	–	–	(+)	–
5	Anis B, 2013, IJD	47/M	Middle of the chest	(+)	–	(+)	–	T2N3M1	IV	–	(−)	–	–	–	–	(−)	–	–	–	–	–	–	–	–	–	(+)
6	Reyes CV, 2008, Pacing Clin Electrophysiol	88/F	Right lateral chest	(−)	Dead [24]	(−)	–	T2N0M0	II	MG	–	–	–	(+)	–	–	20%	(−)	–	(+)	(+)	–	–	–	(−)	–
7	Nash JW, 2007, J Cutan Pathol	44/M	Right chest	(+)	Alive [several]	(+)	H	T2N2M0	IV	CT, LUS	–	(+)	(+)	(+)	–	–	–	(−)	(+)	–	(+)	(+)	(+)	(+)	(+)	–
8	Long WP, 1998, Dermatol Surg	66/F	Above the sternum	(−)	Alive [18]	(−)	L	T4N0M0	IV	–	–	(+)	–	–	(−)	–	–	(−)	–	(−)	(−)	–	–	–	–	(+)
9	Waxtein L, 1998, Int J Dermatol	54/M	The sternal area of the chest	(−)	–	(+)	L	T1N1/2MO	III/IV	–	(+)	–	–	–	(+)	(+)	–	(+)	–	–	–	–	–	–	–	–

IHC, immunohistochemistry; DG, differentiation grade; AE, auxiliary examination; CK, cytokeratin; CK-HMW, human high molecular weight cytokeratin; CK5, cytokeratin 5; CK7, cytokeratin 7; EMA, epithelial membrane antigen; CEA, carcinoembryonic antigen; Ki-67, Ki-67 antigen; S100, S100 protein; AR, androgen receptor; PR, progesterone receptor; ER, estrogen receptor; EGFR, epidermal growth factor receptor; HER-2/neu, human epidermal growth factor receptor 2; p63, tumor protein p63; p53, tumor protein p53; PAS, periodic acid-Schiff; F, female; M, male; L, low grade; H, high grade; (+), positive result; (−), negative result; –, not reported; HFUS, high-frequency ultrasound; CDFI, color Doppler flow imaging; CEUS, contrast-enhanced ultrasound; MG, mammography; LUS, liver ultrasound.

No difference was observed in the proportions of men and women. Except for one patient who developed the disease at a young age due to DICER1 tumor predisposition syndrome (8), the remaining patients were older than 40 years. Furthermore, almost all patients had no obvious symptoms. The diagnosis of HAC of the chest wall primarily relied on the results of immunohistochemical analysis. In our statistics, only three case reports conducted imaging examinations, primarily ultrasound, CT, and X-ray. However, no previous studies reported the use of PET/CT. These imaging modalities are useful for staging purposes but do not provide a reliable preliminary diagnosis prior to pathological examination. Immunohistochemical analysis showed high positivity rates for CK, human high molecular weight cytokeratin, CK7, CEA, Ki-67, and estrogen receptor. Among the patients examined, four had axillary lymph node metastasis in the chest wall HAC, three developed axillary lymph node metastasis after the initial surgery, one had preoperative lymph node metastasis (a patient with high-grade HAC), and two of these four patients also had concurrent abdominal wall metastasis. The lymphatic and distant metastasis rates of chest wall HAC are approximately 50% and 25%, respectively. This finding underscores the highly aggressive and metastatic nature of HAC. Notably, patients with high-grade HAC of the chest wall appear to be more susceptible to metastasis, as demonstrated in our reported case. Among the observed patients, four with low-grade HAC showed the absence of metastases, while the only patient with high-grade HAC presented with stage IV lymphatic metastases, indicating the highly invasive and metastatic nature of HAC (3).

Historically, wide local excision (WLE) was the predominant treatment for HAC of the chest wall. Our statistical literature review found that seven of eight patients with metastasis had undergone WLE, with three developing metastasis after surgery, resulting in a postoperative metastasis rate of 42%. The most recent patient underwent Mohs microsurgical protocol (MMS), and no recurrence or metastasis was observed during follow-up.

To explore the relationship between stage and prognosis, we staged the patients according to the descriptions in the case report. As no separate staging system is available for HAC, the staging method used for skin squamous cell carcinoma recommended by the American Joint Committee on Cancer is still applied. However, of the five patients who underwent follow-up, only one died (due to sudden coronary thrombosis), while the remaining four patients were followed up for 5 months to 2 years. Despite the short follow-up period, the prognosis of chest wall HAC appeared favorable, as none of the patients died from the disease, particularly among those who underwent early surgery. This finding aligns with the observations of Teng Gao et al. regarding the prognosis of HAC (6). Stage did not appear to be associated with prognosis. Although this may be due to the small sample size or inaccuracies in staging, a recent statistical analysis found no significant association between the two (6).

## 4 Discussion

HAC has the same histological features as most epithelial tumors, including hypercellularity and pleomorphic cells. Although most patients present with HAC *de-novo* cases, some develop HAC due to the malignant transformations of benign hidradenomas (16). The distinction between metastatic adenocarcinoma and primary HAC can be difficult (17), and its differential diagnosis is particularly challenging. Currently, the primary diagnostic tools for HAC are histopathology and immunohistochemistry, completed by imaging examinations such as PET/CT (18).

HAC showed strong expression of p63, CK5/6, and CK but negative expression of S100 and smooth muscle actin. EMA and CEA expressions were positive. Additionally, the Ki-67 and phosphohistone H3 labeling indices and tumor protein p53 (p53) expression levels were higher in patients with malignant hidradenoma (19). However, in our literature review of HAC of the chest wall, an equal number of patients tested positive and negative for EMA. Consistent with our findings, some scholars have reported variable expression of CEA and EMA in patients with HAC of the chest wall (10). The expression level of Ki-67, which is closely related to cell proliferation and growth, is widely used as a tumor proliferation marker in pathological examinations (20). The progressive growth of Ki-67 is more common in patients with HAC, with our patient exhibiting a Ki-67 level of 80% (21). Skin adnexal tumors are classified into high-grade and low-grade variants, which affect prognosis (7). The patient in our report is the second documented individual with high-grade HAC of the chest wall demonstrating extensive metastases.

^18^F-FDG PET/CT is an emerging imaging technique that can be used to identify and detect metabolic changes that precede structural changes in tissues and organs at the molecular level. PET imaging reflects the metabolic activity of tumor cells by measuring the uptake and accumulation of ^18^F-FDG in tumor tissues, thus providing a reliable basis for the early detection of lesions and clinical staging. Additionally, fused CT images can further enhance the anatomical images and improve the accuracy of localization (22). ^18^F-FDG PET/CT is highly valuable for revealing the details of tumor metabolism and detecting distant metastasis, with high sensitivity for the detection of primary sites and metastases (23). For HAC, a highly invasive and metastasis-prone malignant tumor, ^18^F-FDG PET/CT is an excellent tool for initial staging, monitoring metastasis, and following up patients (24). Recent literature reports on ^18^F-FDG PET/CT performed in patients with HAC have highlighted cases involving the knee joint (SUVmax: 20.0), head and neck (SUVmax: 22.4), and abdominal wall. Our ^18^F-FDG PET/CT imaging of HAC in the chest wall (SUVmax: 23.8) further contributes to this body of work (24–26). Hypermetabolism is commonly observed in sweat gland malignancies and their metastatic lesions. This effect is more pronounced in HAC, with the SUVmax reaching 20. ^18^F-FDG PET/CT is also used in detecting *in-vivo* tumor metastasis in patients with abnormal skin tumors, particularly when multiple lesions appear on the body surface. Hence, it serves as a valuable tool for distinguishing between metastatic adenocarcinoma and primary HAC (27, 28). Skin metastasis from internal malignancies may yield a false- positive result although the incidence is low. Therefore, ^18^F-FDG PET/CT examination of HAC can not only explore its invasion range, lymphatic metastasis, or distant metastasis but also differentiate primary HAC from other metastatic tumors. However, ^18^F-FDG uptake may not be significantly elevated in early-stage HAC, possibly due to only mildly increased glucose metabolism at this stage. In such cases, imaging with ^68^Ga-fibroblast activation protein inhibitor (FAPI) PET/CT can be considered, as intense ^68^Ga-FAPI uptake has been reported in HAC (29). This highlights the potential use of PET/CT in the diagnostic evaluation of HAC.

Given the rarity of HAC, the optimal treatment remains unclear, and the effectiveness of chemoradiotherapy is controversial (30, 31). In this case, early surgical resection followed by adjuvant therapy is commonly used. This approach may improve the prognosis of HAC and reduce the risk of recurrence (26). WLE with or without lymph node dissection appears to be a relatively common surgical treatment for HAC (10). Two-step surgery can facilitate better control of margin quality and achieve satisfactory control through MMS (32, 33). Chemoradiotherapy is not routinely used in the treatment of HAC. According to the literature, chemoradiotherapy is performed in only 0.4% of patients, typically reserved for those with inoperable cases or a strong tendency for metastasis and invasion (34).

## 5 Conclusion

This report presents a rare case of high-grade HAC of the chest wall and its ^18^F-FDG PET/CT images, a literature review, and a discussion on the relevant immunohistochemical examination and treatment. We found that the chest wall HAC tends to metastasize although the prognosis may not be poor, particularly when diagnosed early and treated with surgery. Unfortunately, the patient refused treatment after developing metastasis, highlighting the importance of early diagnosis. Therefore, combining PET/CT with pathological examination can enhance diagnostic efficiency, improve staging, enable early intervention, and ultimately improve prognosis.

## Data Availability

The original contributions presented in the study are included in the article/Supplementary Material. Further inquiries can be directed to the corresponding author.
